# *Leishmania* (*Leishmania*) *infantum* and *Leishmania* (*Sauroleishmania*) *tarentolae* in outdoor cats and report of infection in feline-derived peripheral blood mononuclear cells

**DOI:** 10.1186/s13071-025-06983-w

**Published:** 2025-08-26

**Authors:** Jairo Alfonso Mendoza-Roldan, Mariaelisa Carbonara, Viviane Noll Louzada-Flores, Mario H. Alves, Nicola Pugliese, Nicola Decaro, Annamaria Uva, Floriana Gernone, Maria Alfonsa Cavalera, Andrea Zatelli, Domenico Otranto

**Affiliations:** 1 Department of Veterinary Medicine, University of Bari, Bari, Italy; 2 Department of Veterinary Clinical Sciences, City University of Hong Kong, Hong Kong, China

**Keywords:** *Leishmania infantum*, *Leishmania tarentolae*, Cat, Feline leishmaniosis, Experimental infection

## Abstract

**Background:**

Feline leishmaniosis (FeL) is mainly caused by *Leishmania infantum* in the Mediterranean Basin. In Italy, in the same epidemiological context where canine leishmaniosis (CanL) is hyperendemic, a nonpathogenic species, *Leishmania tarentolae*, may also occur in sympatry, infecting reptiles, dogs, and humans. Thus, this study aimed to assess *L. tarentolae* infection in outdoor cats along with its co-occurrence with *L. infantum* and to evaluate risk factors. In addition, the persistence of *L. tarentolae* in feline-derived peripheral blood mononuclear cells (PBMCs) was herein evaluated in vitro.

**Methods:**

Outdoor colony or stray cats were screened for *Leishmania* spp. by immunofluorescence antibody test (IFAT) using promastigotes of both *L. infantum* and *L. tarentolae*. Whole blood and buffy coat were tested by a real-time polymerase chain reaction (qPCR) and duplex real-time PCR (dqPCR), and positive samples sequenced following an ITS1 conventional PCR (cPCR). Feline-derived PMBCs were subsequently infected with promastigotes of *L. tarentolae* to assess the persistence of amastigotes. Viral infections caused by feline immunodeficiency virus (FIV) and feline leukemia virus (FeLV) were molecularly addressed in all enrolled cats. Statistical analysis was performed to evaluate the possible association between *Leishmania* spp. infection and FIV/FeLV infection by using a multivariate logistic regression model following an initial LASSO-penalized logistic regression.

**Results:**

Overall, 42 out of 194 cats (21.6%) were serologically or molecularly positive for *Leishmania* spp. In particular, 26 (13.4%) cats were seropositive for *L. infantum* and/or *L. tarentolae* by IFAT, with 16 (8.2%) animals positive for both species. Molecularly, 14 out of 194 cats (7.2%) were positive for *L. infantum* by qPCR, whereas five (2.6%) were positive for *L. tarentolae* by dqPCR. Cat PBMCs were successfully infected with *L. tarentolae,* and the infection persisted for at least 72 h. Overall, 38 out of the 194 screened cats (19.6%) were infected by FIV and/or FeLV, of which 12 were serologically or molecularly positive for *Leishmania* spp., with one cat positive for *L. tarentolae* DNA, and five for *L. infantum* DNA. Multivariate screening identified municipality (OR 2.206; *P* = 0.031; 95% CI 1.077–4.516) as a risk factor for *Leishmania* spp. infection, while the association between *Leishmania* spp. and FIV infection was not significant (OR 2.359; *P* = 0.08, 95% CI 0.901–6.179).

**Conclusions:**

Colony or stray cats were herein found for the first time infected by *L. tarentolae*, in areas where *L. infantum* is endemic. Cross-reactivity using IFAT test may pose a diagnostic hindrance also in FeL. The infection with this saurian-associated *Leishmania* in cats was further confirmed through the persistence of this *Leishmania* in cat PBMCs. Further studies are needed to fully unravel the complex interactions between both species of *Leishmania* and the implication of the sympatric occurrence of both species in the diagnosis and control of leishmaniosis.

**Graphical Abstract:**

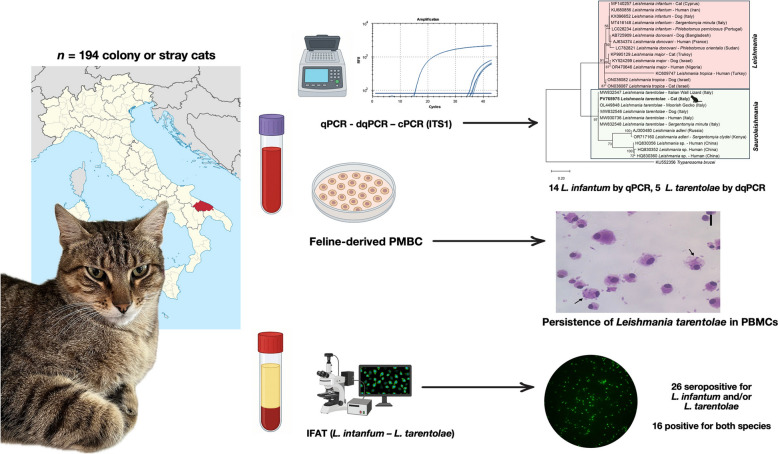

## Background

*Leishmania* (Kinetoplastida: Trypanosomatidae) is a group of protozoan parasites vectored mainly by phlebotomine sand flies (Diptera: Psychodidae), that feed on birds, mammals, and reptiles, the latter two representing the natural vertebrate hosts [1, 2]. The genus *Leishmania* is further divided in four subgenera, of which *Leishmania* and *Sauroleishmania* are distributed mostly in the Old World, while *Viannia* is spread in the New World, and *Mundinia* in the Australasian ecoregion [1, 3]. Most of the species in the subgenera mentioned above, including *Sauroleishmania*, may also infect humans, and are of rising zoonotic concern [3, 4].

In the European Mediterranean Basin, *Leishmania infantum* is the main species present, which infects mainly dogs, and is the causative agent of canine leishmaniosis (CanL) [5]. Nonetheless, apart from human infections, domestic and wild animals, both mammals and reptiles, may contribute to the circulation of the parasite [6, 7]. For example, lagomorphs (i.e., rabbits and hares) as well as cats may play an epidemiological role, particularly in the absence of the primary canine host [6, 8]. Cats are of significance as reservoirs for *L. infantum* due to their close proximity to humans and their outdoor lifestyle [9]. For instance, a serological prevalence of 17% for *Leishmania* spp. was identified across six Mediterranean countries, namely Portugal, Greece, Israel, Spain, France, and Italy [10]. Nevertheless, cats are less susceptible to develop the disease than dogs, with only a few animals presenting cutaneous and mucocutaneous lesions (e.g., ulcers and nodules), lymphadenomegaly, ocular lesions (mainly uveitis), or feline chronic gingivostomatitis syndrome [11]. Indeed, the seroprevalence of *L. infantum* in cats in endemic regions is roughly half of that of dogs [12]. Risk factors for feline leishmaniosis (FeL) include an outdoor lifestyle (i.e., exposure to sand fly bites) and viral co-infections with feline immunosuppressive and/or leukemia viruses (i.e., FIV, FeLV), along with treatment with corticosteroids [10, 13, 14].

Furthermore, cats may also be infected by other species of *Leishmania* from the *Leishmania* subgenus, such as *Leishmania tropica, Leishmania major*, as well as a putative hybrid of *Leishmania major* and *Leishmania donovani* sensu lato in Portugal [15] and *Leishmania braziliensis*, and *Leishmania guyanensis* (*Viannia* subgenus) in South America [15, 16].

In Italy, *L. infantum* is widely distributed, with hyperendemic areas of CanL in the southern regions [17], where cats [14] and other wild felids (e.g., tigers) [18] may also be infected. However, other species of *Leishmania* are also present in Italy, such as a putative anthroponotic hybrid of *L. infantum* and *L. donovani* in the Northern Emilia-Romagna region [19], and the nonpathogenic saurian-associated *Sauroleishmania, Leishmania tarentolae* [20]. This latter species has been detected in Squamata reptiles worldwide, and in southern Italy [7, 21] with reports in dogs [22] and humans [23, 24] as well. Importantly, this saurian-associated species of *Leishmania* may persist and elicit a Th1 “protective” phenotype (i.e., cellular response) in canine-derived peripheral blood mononuclear cells (PBMCs) [25, 26], as well as in the canine model [27]. This picture suggested that *L. tarentolae* could potentially reduce the risk of disease onset by exploiting its cross-immunity with *L. infantum* [20]. Conversely, the co-occurrence of both species of *Leishmania* in the Italian territory may pose diagnostic challenges, especially when it comes to the performance of serological tests routinely used in clinical practice, such as indirect immunofluorescence test (IFAT) [22, 28]. Specifically, IFAT has several limitations in terms of diagnosis, which are linked not only to the subjective interpretation of fluorescence levels, but also to cross-reactions that may occur within the *Leishmania* genus [9, 28]. When more than one species of *Leishmania* is present in a given area, enzyme-linked immunosorbent assay (ELISA) using recombinant antigens should be preferred over IFAT for clinical diagnosis. This is due to the fact that ELISA, utilizing species-specific recombinant antigens, has greater specificity and reduced subjectivity compared with other methods [28].

Given the paucity of information regarding FeL, its endemicity, and potential infectivity of *L. tarentolae* in cats, the aim of the present study was to identify *L. tarentolae* in outdoor cats, assessing its co-occurrence with *L. infantum* and evaluating risk factors. Furthermore, the persistence of *L. tarentolae* in feline-derived PBMCs was also assessed under laboratory conditions to corroborate field data and provide basic scientific information about the cellular interaction of this protozoon in cats.

## Methods

### Sample collection

From July 2022 to July 2024, stray or colony cats were referred to the Veterinary Teaching Hospital of the University of Bari “Aldo Moro” (Bari, Italy) for evaluation and management of diverse clinical presentations, under the frame of an agreement between the municipalities of Bari (site A) and Valenzano (site B) (authorization D.R. n. 4229 of 18/11/2022, art. 2 and 11). Both sites are known to be endemic for CanL [17] as well as for sand fly vectors (i.e., mainly *Phlebotomus perniciosus* and *Sergentomyia minuta*) [22]. At enrollment, signalment data (i.e., age, sex, reproductive status) and the geographical area (i.e., zip code, city/town) for each cat were recorded in individual files. Age was categorized as young, pre-adult, and adult on the basis of information provided by the volunteers responsible for the animals. In cases of substantial discrepancies or uncertainty, age estimation was performed by the attending veterinarians by teeth evaluation. All animals underwent a complete clinical examination and laboratory testing as part of their routine diagnostic workup. Specifically, on the basis of the convenience of sample availability, an aliquot of each biological sample collected within the routine diagnostic screening was also analyzed by the Parasitology Unit of the Department of Veterinary Medicine, University of Bari (Bari, Italy)for the detection of *Leishmania* spp., as detailed below. Additionally, for each animal, blood smears were prepared and stained using Diff-Quik® (RAL Diagnostics, Martillac, France) to assess the presence of *Leishmania* parasites. After staining, the smears were rinsed with tap water to remove excess dye and subsequently examined under an optical microscope (Zeiss Axioscope 5, Germany). For the cat PBMC preparation and infection assays, the study protocol was approved by the ethical committee of the Department of Veterinary Medicine of the University of Bari, Italy (Prot. Uniba 56/2024). The methods were carried out in accordance with international, national, and/or institutional guidelines and regulations for handling animal cells.

### Serology

Cat serum samples were tested by IFAT for the detection of immunoglobulin (Ig)G anti-*L*. *infantum* and *L*. *tarentolae*, following the same procedure previously described [24, 29]. For IFAT, serum samples from a cat that tested positive for *L. infantum* by molecular analysis and a negative healthy cat were used as positive and negative controls, respectively. Though the IFAT test for *L. tarentolae* was not fully validated, given the lack of *L. tarentolae*-positive sera from cats, the samples were scored as positive when they produced clear cytoplasmic and membrane fluorescence of promastigotes from a cutoff dilution of 1:80 [10, 29]. Positive sera were titrated by serial dilutions until negative results were obtained.

### Molecular screening

Genomic DNA (gDNA) was extracted from cat whole blood (200 μl) and buffy coat samples by using commercial kit (QIAamp DNA Mini Kit, Qiagen, Hilden, Germany), according to the manufacturer’s instructions. All extracted samples were tested for the detection of FIV and FeLV proviral DNA by PCR using primers and protocol described previously [30, 31], as well as by using a duplex real-time PCR (dqPCR) for the detection of partial region of the internal transcribed spacer 1 (ITS1) gene of *L*. *infantum* and *L*. *tarentolae*, as described elsewhere [32]. Moreover, real-time PCR (qPCR) for the detection of *L*. *infantum* kDNA minicircle (120 bp) by using primers, probes, and protocol described elsewhere were also used for all samples [33]. Genomic DNA from *L*. *infantum* isolate cultured in Tobie-Evans medium from a dog with leishmaniosis living in Italy (zymodeme MON-1) and *L*. *tarentolae* (strain RTAR/IT/81/ISS21-G.6c/LEM124) promastigotes were used as positive controls, whereas gDNA extracted from blood sample of a healthy cat negative for *L*. *infantum* was used as negative control, as previously described [22, 24]. For sequence analysis, *Leishmania* qPCR and dqPCR-positive samples were amplified by conventional PCR (cPCR) using primers L5.8S/LITSR targeting the ITS1 gene (~300 bp) and PCR protocol run as described elsewhere [34]. To discard the presence of morulae of *Anaplasma* spp. or *Ehrlichia* spp., positive samples identified through *Leishmania* qPCR and dqPCR were further analyzed using cPCR with primers EHR16SD/EHR15SR, targeting the 16S rRNA gene of Anaplasmataceae (345 bp) [35], to rule out any alternative explanations of intracytoplasmic inclusions. Amplicons were purified and sequenced in both directions using the same primers as for PCR, employing the Big Dye Terminator v.3.1 chemistry in an automated sequencer (3130 Genetic Analyzer, Applied Biosystems, Foster City, CA, USA). Samples were compared with those available in GenBank using the BLASTn tool (http://blast.ncbi.nlm.nih.gov/Blast.cgi).

To determine genetic clustering of *L. tarentolae*, the representative ITS1 sequences obtained from cat samples and from reference strains of *L. tarentolae* and *L. infantum* were phylogenetically analyzed along with those of other *Leishmania* spp. available in the GenBank database. Selected sequences (*n* = 26) were aligned using ClustalW [36] (using pairwise and multiple alignments with a gap opening penalty of 15.00, and a gap extension penalty of 6.66) included in the software MEGA v.12. Phylogenetic relationships were inferred using the maximum likelihood (ML) method on the basis of the Tamura 3-parameter model [37], and discrete gamma distribution with invariants sites (G+I) was used to model evolutionary rate differences among sites, selected by best-fit model analysis and on the basis of the lowest score obtained by Bayesian information criterion (BIC) using MEGA12 software [37]. Evolutionary analyses were conducted with 2000 bootstrap replications using MEGA12 software [38]. The corresponding ITS1 sequence of *Trypanosoma brucei* (GenBank: KU552356.1) was used as outgroup.

### Cat PBMC infection with *Leishmania tarentolae*

#### Strains and cultures

*Leishmania tarentolae* strain isolated from *Tarentola mauritanica* gecko (RTAR/IT/22/R011), at its sixth passage, was cultivated in Schneider’s *Drosophila* medium (ThermoFisher Scientific, USA) supplemented with 10% fetal bovine serum (FBS) and maintained at 26 °C for 5 days prior to cell infection.

#### Cat PBMC preparation and infection assays

A total of 4 mL of blood was collected from a healthy cat into ethylene diamine tetra acetic acid (EDTA) tubes (BD Vacutainer, San Diego, CA, USA). Specifically, the cat used for PBMC isolation was a privately owned animal. A blood sample of this animal was sent to the *Istituto Zooprofilattico Sperimentale delle Venezie* (IZSVe) for diagnostic screening of major feline pathogens, including *Leishmania* spp., and tested negative. Furthermore, the animal was subjected to a thorough clinical evaluation. No clinical signs or laboratory alterations were detected (i.e., hematology and biochemistry results were unremarkable). Peripheral blood mononuclear cells were isolated following the procedure described by Louzada-Flores et al [25], with modifications. Briefly, the blood was mixed in equal volumes with RPMI-1640 medium (Euroclone, Milan, Italy), and subsequently layered over twice the volume of Lympholyte®-H (Cedarlane, Italy), followed by centrifugation at 400 × g for 45 min at room temperature (RT). The resulting buffy coat was collected and washed twice with pre-warmed RPMI-1640 medium, and centrifuged at 310 × g and then 210 × g for 10 min each at RT. The recovered PBMCs were resuspended in warm RPMI-1640 medium supplemented with 10% FBS, a 1% penicillin–streptomycin solution (100×), and 50 ng/mL of human recombinant CSF-1 (Proteintech). Cells were seeded at a density of 2 × 10^5^ cells/mL into 24-well plates and incubated for 5 days. On day 5, cells were infected with the *L. tarentolae* strain at a ratio of 10 parasites per cell. Specifically, promastigotes of *L. tarentolae* (strain R011) were collected on day 5 of stationary-phase culture (i.e., the timepoint with the highest proportion of metacyclic forms for this strain) and were used as *inoculum* at a ratio of 10:1 parasite per cell. The percentage of infected cells and the number of parasites per infected cell were assessed 72 h post-infection.

### Statistical analysis

The sample size was calculated using Cochran’s formula [39], with a correction for a finite population [40] based on the assumptions of an expected prevalence of 25.8% in endemic areas of southern Italy [12], 5% precision, and 95% confidence interval, with a finite population of 300 cats. On the basis of those parameters, a minimum sample size of 149 subjects was determined, then increased by 30% due to the reported underestimation of the correction for the finite population. Potential risk factors for *L. infantum* and *L. tarentolae* infection, or both, were preliminarily analyzed by performing an initial LASSO-penalized logistic regression with tenfold cross-validation. Briefly, a design matrix of all the candidate predictors was constructed and analyzed by specifying a binomial distribution of errors. The penalty parameter *λ*, which minimized the mean cross-validated deviance (*λ*_min_), was identified and variables with nonzero coefficients at *λ*_min_ were retained. The identified candidate variables were incorporated into a multivariable logistic regression model, with adjusted odds ratios (OR) with 95% confidence intervals (CI) [41]. The area under the receiver‐operating characteristic (ROC) curve (AUC) and the maximized log-likelihood were assessed, as well as the model calibration, was checked by the Hosmer–Lemeshow goodness‐of‐fit test. ORs were considered significant when *P* < 0.05. All analyses were performed in R version 4.5.0 (R Foundation for Statistical Computing, Vienna, Austria) using glmnet [41], pROC [42], and ResourceSelection [43].

## Results

Of the 194 cats screened from both sites, 42 (21.6%; Table 1) were serologically or molecularly positive for *Leishmania* spp., with prevalence ranging from 15.2% (site B) to 29.2% (site A). *Leishmania* spp.-positive animals were referred to the veterinary hospital due to trauma, depression, or stomatitis, with only one presenting skin lesions (site B, cat number 1), which was also molecularly positive for *L. infantum* (Table 1).

**Table 1:** Signalment data (i.e., age, sex, reproductive status, and municipality), viral infections, and reason for referral to veterinary hospital of the 42 outdoor cats tested serologically (IFAT) and/or molecularly (qPCR, dpPCR) positive for *Leishmania* spp.

Cat ID	Age (>1 year; <1 year)	Sex	Reproductive *status*	Municipality	IFAT *L. infantum*	IFAT *L. tarentolae*	qPCR (*ct*—tissue)	dqPCR (*ct, Leishmania* sp.—tissue)	Viral infection	Reason for referral
04	>1 year	F	Not neutered	Bari	1:80	–	–	–	–	Respiratory signs
05	>1 year	M	Neutered	Bari	1:80	–	32—BC	–	FeLV	Nasal and conjunctival discharge
06	<1 year	M	Not neutered	Bari	–	–	–	38, *L. tarentolae*—BC	–	Depression
22	>1 year	M	Not neutered	Bari	1:80	–	–	–	FIV/ FeLV	Mucopurulent nasal discharge
23	>1 year	M	Not neutered	Bari	–	–	32—BC	–	–	Trauma
31	>1 year	F	Not neutered	Bari	–	–	32—BC	–	–	Trauma
37	>1 year	M	Neutered	Bari	–	–	–	35, *L. tarentolae*—WB	–	Severe stomatogingivitis
40	<1 year	F	Not neutered	Bari	1:160	1:80	–	–	–	Pyelonephritis
41	>1 year	M	Not neutered	Bari	–	–	28—BC	–	–	Trauma fracture
42	>1 year	M	Neutered	Bari	1:160	–	36—WB	–	FIV	–
43	>1 year	M	Not neutered	Bari	1:80	1:80	–	–	FIV	Right hemothorax
44	>1 year	F	Not neutered	Bari	–	–	36—WB	–	–	–
47	>1 year	M	Not neutered	Bari	1:160	1:80		–	–	Stomatitis
48	>1 year	F	Not neutered	Bari	1:160	–	–	–	–	Otohematoma
50	>1 year	M	Not neutered	Bari	1:320	1:160	–	–	–	–
61	>1 year	M	Not neutered	Bari	1:80	1:80	–	–	–	–
64	>1 year	F	Not neutered	Bari	–	1:80	–	–	–	Bilateral ocular discharge
71	>1 year	F	Neutered	Bari	1:160	1:80	–	–	–	Gingivostomatitis
72	>1 year	F	Neutered	Bari	1:80	–	–	–	–	–
79	>1 year	M	Not neutered	Bari	1:320	1:160	–	–	FIV	Nasal discharge
85	>1 year	M	Not neutered	Bari	–	–	–	37, *L. tarentolae*—BC	–	Gait deficit
86	>1 year	M	Not neutered	Bari	–	–	32—BC	–	FeLV	Ataxia
89	>1 year	M	Not neutered	Bari	1:160	1:80	–	–	FIV	Respiratory signs
96	<1 year	F	Not neutered	Bari	1:80	1:80	–	–	–	Gingivostomatitis
97	>1 year	F	Not neutered	Bari	–	–	36—WB	–	–	Verminous emesis
105	>1 year	F	Not neutered	Bari	1:80	1:80	–	–	–	Gingivostomatitis
1	>1 year	F	Not neutered	Valenzano	–	–	32—WB	–	–	Skin lesions
21	<1 year	F	Not neutered	Valenzano	1:80	1:160	–	–	–	Lameness
30	>1 year	F	Not neutered	Valenzano	–	–	–	37, *L. tarentolae*—WB	FIV	Respiratory signs
41	<1 year	F	Not neutered	Valenzano	–	–	–	37, *L. tarentolae*—WB	–	Respiratory signs
44	>1 year	F	Not neutered	Valenzano	–	–	36—WB	–	FeLV	Trauma
52	>1 year	F	Neutered	Valenzano	–	–	37—WB	–	–	Depression
61	<1 year	M	Neutered	Valenzano	1:80	1:80	–	–	–	Depression
66	>1 year	M	Not neutered	Valenzano	1:160	–	–	–	FIV	Conjunctivitis
68	>1 year	M	Not neutered	Valenzano	1:80	–	–	–	–	Tail amputation
75	>1 year	M	Not neutered	Valenzano	1:80	–	–	–		Depression
78	>1 year	M	Not neutered	Valenzano	1:80	1:80	–	–	–	Stomatitis
89	>1 year	M	Not neutered	Valenzano	1:320	1:80	–	–	FIV	Anorexia
90	>1 year	M	Not neutered	Valenzano	–	–	36—BC	–	FIV	Trauma
100	<1 year	F	Not neutered	Valenzano	1:80	1:80	36—BC	–	–	Trauma
104	>1 year	F	Neutered	Valenzano	–	–	36—BC			
105	>1 year	M	Neutered	Valenzano	1:160	1:160	–	–	–	–

*F* female, *M* male, *WB* whole blood, *BF* buffy coat

Overall, 26 cats (13.4%) scored positive against promastigotes of *L. infantum* and/or *L. tarentolae* by IFAT (Table 1) of which 9 (4.6%) only against promastigotes of *L. infantum*, and 1 of *L. tarentolae* (0.5%). On the contrary, the remaining 16 cats (8.2%) were positive for both IFAT tests with different titrations (Table 1).

Molecularly, 14 animals (6.6%) scored positive for *L. infantum* through qPCR (mean *Ct* value 34.08 ± 2.77; median *Ct* value 36) on whole blood (*n* = 6) and buffy coat (*n* = 8), with three also positive by IFAT (Table 1). In addition, *L. tarentolae* was molecularly detected in five seronegative cats (2.4%) through dqPCR in whole blood (*n* = 3) and buffy coat (*n* = 2) (mean *Ct* value 36.33 ± 1.154; median *Ct* value 37). When compared, mean and median *Ct* values were lower in buffy coat (i.e., mean *Ct* value 33 ± 2.6; median *Ct* value 32) than in whole blood (i.e., mean *Ct* value 35.6 ± 1.69; median *Ct* value 36) for *L. infantum*, whereas whole blood had lower mean and median *Ct* values (i.e., mean *Ct* value 36.3 ± 0.94; median *Ct* value 37) than buffy coat (i.e., mean *Ct* value 37.5 ± 0.5; median *Ct* value 37.5) for *L. tarentolae.*

**Fig 1.**
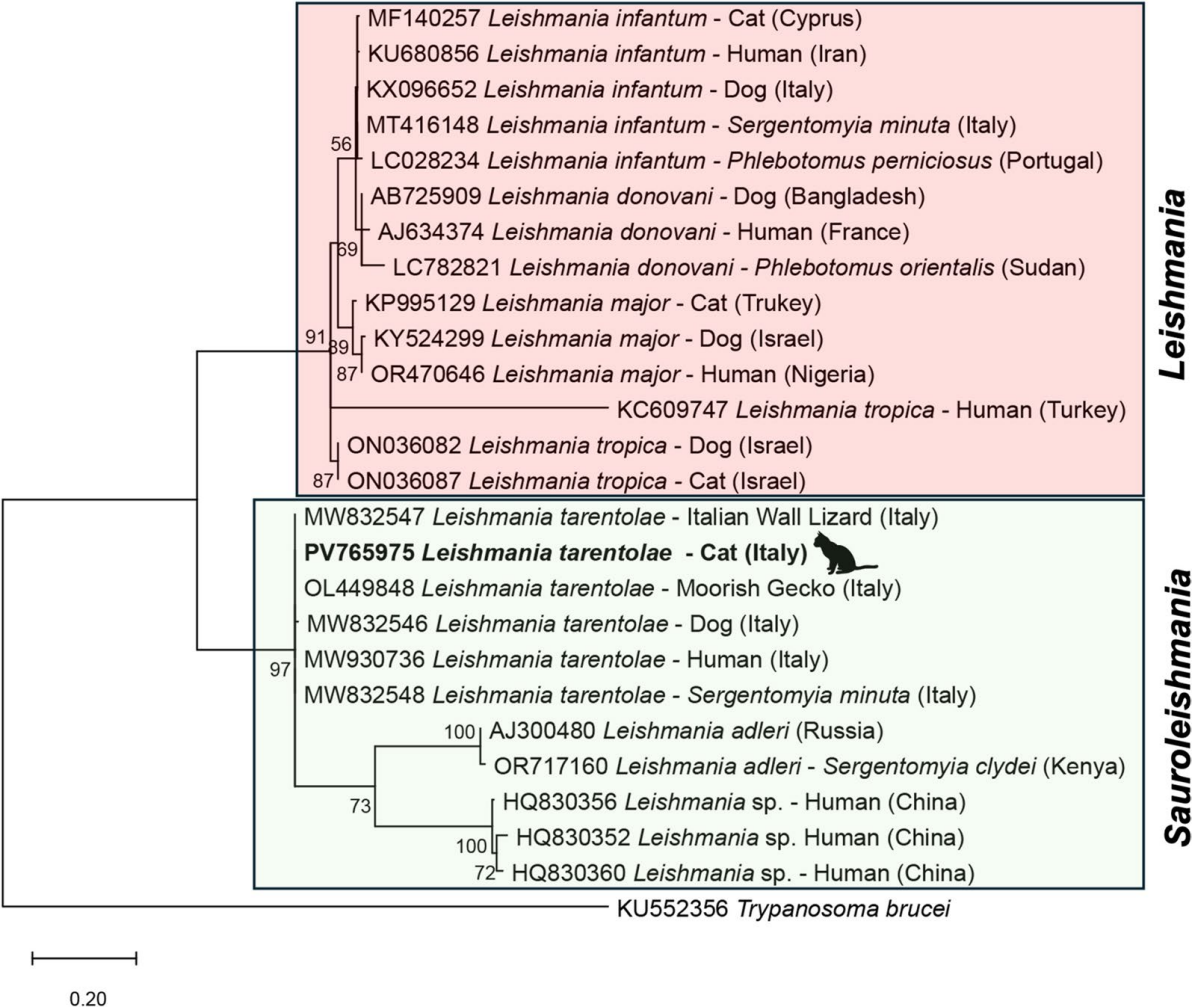
Phylogenetic tree based on *Leishmania* ITS1 sequences inferred using the maximum likelihood method on the basis of Kimura 2-parameter model. Bootstrap values (>60%) are shown near the nodes. *Trypanosoma brucei* is used as outgroups. Scale bar indicates nucleotide substitution per site. *Leishmania* spp. sequenced in this study are in bold

Given the high *Ct* values, ITS1 sequences were only obtained from a cat (number 37; *Ct* value 35) that was deposited in GenBank for *L. tarentolae* (accession number PV765975). Blast analysis of ITS1 sequence of *L. tarentolae* showed a 100% nucleotide identity with reference sequences deposited in GenBank, derived from a *S. minuta* sand fly collected in central Italy (accession number MT416142), from *Tarentola mauritanica* from southern Italy (accession number OL449848), and *Podarcis siculus* from southern Italy (accession number MW832547). Accordingly, the phylogram of the ITS1 sequences above confirmed a close phylogenetic relationship among the *L. tarentolae* sequence herein obtained with those from reptiles, mammals, and sand flies by clustering them in a species-specific clade (i.e., *Sauroleishmania*; bootstrap value 97), clearly separated from that of species within the subgenus *Leishmania* (Fig. 1).

Furthermore, a cat referred from site A due to a severe stomatitis tested seronegative but was whole blood dqPCR-positive for *L. tarentolae* and had cytoplasmic inclusions within mature and band neutrophils. This cat was negative for cPCR for Anaplasmataceae, yet cytoplasmatic inclusions were not morphologically compatible with amastigotes. In addition, ITS1 sequence was obtained from this cat and used for the phylogenetic analyses as above (Table 1; cat 37).

In total, 38 cats (19.6%) were infected with FIV and/or FeLV, of which 12 were serologically or molecularly positive for *Leishmania* spp. Specifically, of the *Leishmania* spp.-positive cats that were co-infected by FIV and/or FeLV, one animal was positive for *L. tarentolae* DNA but seronegative, and five for *L. infantum* DNA (i.e., two seropositive for *L. infantum* and three seronegative).

**Fig 2.**
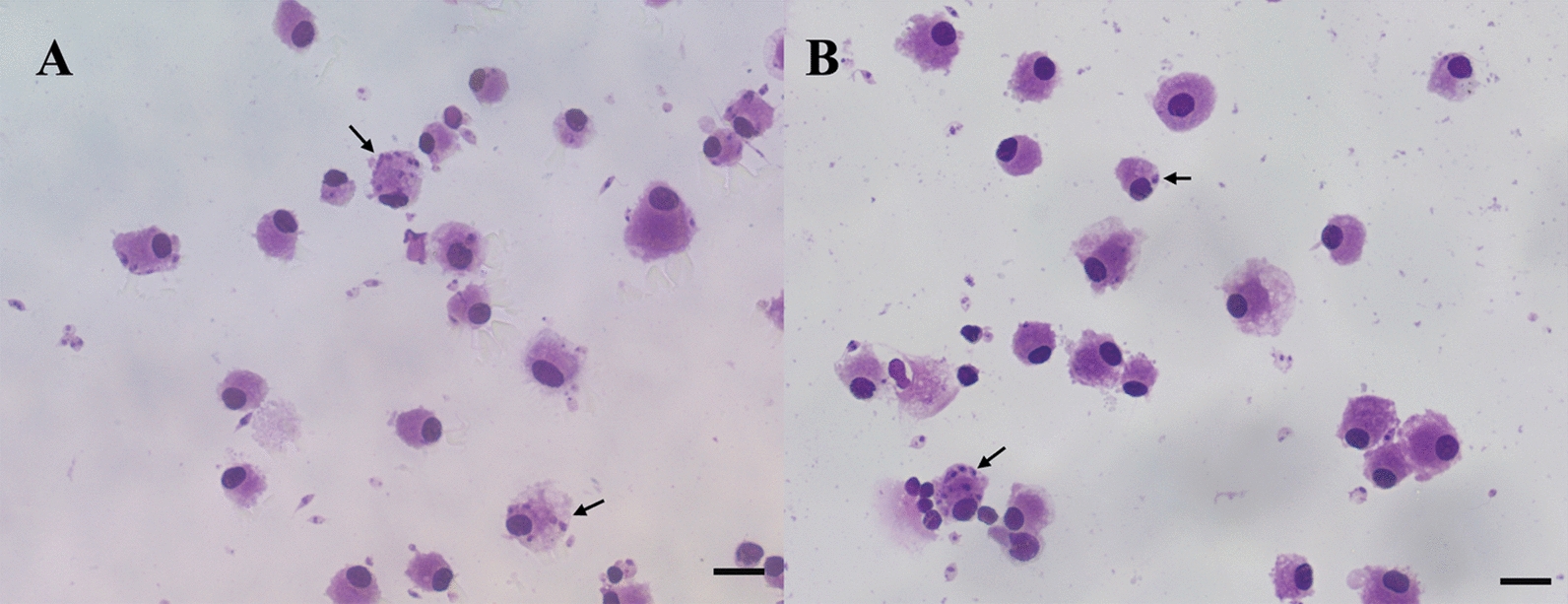
Diff-Quik smears of primary feline monocytic-derived macrophage cells infected by L. tarentolae (RTAR/IT/22/R011), after 72 h of infection. (A - B) amastigotes within macrophage

Cat PBMCs were successfully infected with *L. tarentolae* and the infection was sustained for at least 72 h post-infection (Fig. 2). At that timepoint, the rate of infected macrophages was 41.6%, with an average of 2.1 parasites per infected cell.

The OR was calculated for FIV infection as a potential risk factor (OR 2.359; 95% CI 0.901–6.179), but with a *P*-value of 0.081, which is above the 0.050 threshold and therefore not considered statistically significant. The calibration of the final multivariate test was adequate, as the Hosmer–Lemeshow goodness-of-fit test returned a *P* = 0.718 (*χ*^2^ = 1.346, *df* = 3), while AUC and log-likelihood resulted in 0.657 and −95.793, respectively. When *L. tarentolae* and *L. infantum* were examined singly, no significant results were detected. In both cases, the model fit was optimal (*P* = 1), with good discrimination quality (i.e., AUC = 0.828, log-likelihood = −49.232 for *L. tarentolae* model and AUC = 0.850, log-likelihood = −58.654 for *L. infantum* model).

## Discussion

Results from the present study suggest that colony or stray cats with a mostly complete outdoor lifestyle may be exposed to *L. tarentolae* in areas where *L. infantum* is endemic. Moreover, as reported in dogs and humans, cross-reactivity using IFAT test may pose a diagnostic hindrance in FeL as well. Importantly, in this study we demonstrated for the first time the persistence of *L. tarentolae* in feline monocytic-derived macrophage cells under in vitro conditions.

The cumulative serological and molecular prevalence of *L. infantum* in cats herein reported (18.55%) was higher than previous studies from southern Italy (i.e., 10.5% [14]). This difference between overall prevalence may be due to the fact that all animals screened in the present study had a completely outdoor lifestyle, and were therefore most likely not receiving veterinary care or ectoparasite prophylaxis. Accordingly, when stray cats were screened using ELISA, seroprevalence went up to 27.41% [44], confirming that outdoor lifestyle is an important risk factor for FeL due to the higher exposure to sand fly bites [10]. The relevance of sand fly exposure is further supported by the high seroprevalence of anti-sand fly antibodies (40.7%) previously detected in outdoor cats [44]. Furthermore, outdoor lifestyle may also expose animals to retroviral infections such as FIV, which has been associated with a higher likelihood of *L. infantum* infection [10, 11, 14, 15], increasing the chances of *L. infantum* antibody positivity in cats by 2.8 times compared with those uninfected [45]. In this study, the association between *Leishmania* spp. infection and FIV was not statistically significant, even though the *P*-value (0.081) was slightly above the threshold (0.050). This may be due to the small size of the cat cohort, and further studies in the same area could aid in confirming whether FIV is a risk factor for *Leishmania* spp. infection. Consistently, the final multivariable model demonstrated only modest discrimination (AUC = 0.657). However, model calibration was excellent, indicating that the predicted probabilities align with the observed outcomes across risk strata. Together, these results suggest that the model estimates are unbiased, and the current set of predictors does not provide sufficient contrast to distinguish animals at low or high risk. Future studies should explore additional or more discriminative markers, along with a larger sample size. On the contrary, reasons for referral also support the risks of outdoor lifestyle for animals, which may experience trauma, infectious diseases, envenomation, and even transmission of zoonotic parasites through predation on reptiles, birds, and small mammals [46, 47]. Thus, frequent monitoring and neutering programs are warranted to improve the overall health of colony and stray cat populations.

The slightly more frequent detection of *Leishmania* spp. DNA in buffy coat samples rather than in blood (i.e., 10 positive buffy coat samples versus nine positive blood samples) confirms buffy coat as a more sensitive biological sample, as parasites were more concentrated than in EDTA blood [48]. Nonetheless, given the scarcity of information regarding the life cycle of *L. tarentolae* in mammalian hosts, studies should still consider screening both types of samples, as well as other more frequently used for *Leishmania* spp. molecular detection, such as bone marrow, lymph nodes, and conjunctival swabs [15].

Confirmatory isolation efforts are necessary to determine whether *L. tarentolae* transiently infects mammals or if it may persist, replicate, and later become infective to sand flies. On the contrary, the cross-reactivity observed in IFAT was previously recorded in the Pelagie archipelago, where six cats cross-reacted for *L. tarentolae* and *L. infantum* [24]. Given that 16 animals cross-reacted herein, comparative studies between serological tests are recommended in feline medicine to understand their specificity in detecting antibodies against *L. infantum*, as previously done for the diagnosis of CanL [28].

Instead, molecular data highlighted the possibility of infection rather than just exposure in cats, as suggested by positivity to dqPCR of whole blood and buffy coat for *L. tarentolae* in seronegative cats. The latter picture could be due to a transient presence of the parasite, with still no seroconversion, or to a shift to a Th1 response; both conditions above have already been observed in dogs [22, 27]. Furthermore, the results from PBMCs corroborated the capacity of *L. tarentolae* to establish and maintain an infection in feline mononuclear phagocytes under in vitro conditions, underscoring its relevance for subsequent studies investigating host–pathogen interactions, as well as the immune response to *Leishmania* spp. in cats. Thus, the data retrieved herein establish the domestic cat as a potential mammalian host for *L. tarentolae*, with exposure to and further persistent infection. Nonetheless, transmission routes to feline hosts remain unknown. As with other mammalian and reptilian hosts, the potential transmission route is through sand fly bites by *S. minuta*. The latter, although considered a herpetophilic sand fly, may feed on humans and other mammals [49, 50]. In addition, *P. perniciosus*, which is the main vector of *L. infantum* to mammals, may also be a putative vector of *L. tarentolae* under experimental conditions [51]. Conversely, another possible transmission route could be the ingestion of reptiles by cats, the main reservoir hosts of *L. tarentolae* [22]. Indeed, cats are recognized as apex predators, exerting high predatory pressure on small animals, which may also lead to the passive transmission of *L. tarentolae* [46]. However, this last hypothesis would need further confirmatory evidence.

Finally, as observed with other closely related species of *Leishmania* [19], the sympatric occurrence of *L. infantum* and *L. tarentolae* in sand flies, dogs, cats, humans, and reptiles in the specific epidemiological context here studied may result in hybridization events between these two species. This event has been previously recorded in cats from Portugal, where putative hybrids between *L. infantum* and *L. major* were identified [52]. The likelihood of hybridization and thus genetic exchange between the species studied may have major implications for their diagnostics as well as in their pathogenicity, a hypothesis in need of further investigation. Although IFAT for *L. tarentolae* herein used was previously employed with feline sera [24], it is not yet validated for cats, which represents a limitation of the study that should be overcome in future work.

## Conclusions

Data from the present study confirmed the domestic cat as a new host for *L. tarentolae* in an epidemiological context where this species occurs in sympatry with *L. infantum* in a hyperendemic area. Importantly, as for *L. infantum*, outdoor lifestyle represents a key factor for cat infection due to the higher exposure to sand fly bites and the possible transmission through predation on infected reptiles. Moreover, this study provides the first molecular evidence of the saurian-associated *L. tarentolae* persisting in cats, thereby further expanding its range of potential hosts, as observed for *L. infantum.* The latter is supported by the persistence of *L. tarentolae* amastigotes in cat-derived PBMCs. The serological cross-reactivity for both species in cats, as for CanL, may represent a diagnostic hindrance for FeL, if only IFAT is used. Future studies should elucidate the role of the domestic cat in the increasingly complex epidemiology of both *Leishmania* species that occur in sympatry, under the concept of One Health.

## Data Availability

Sequences were deposited in GenBank (accession number: PV765975).
